# Visual adaptation enhances action sound discrimination

**DOI:** 10.3758/s13414-016-1199-z

**Published:** 2016-09-07

**Authors:** Nick E. Barraclough, Steve A. Page, Bruce D. Keefe

**Affiliations:** 1 Department of Psychology, University of York, Heslington, York YO10 5DD UK; 2 University of Hull, Hull, UK

**Keywords:** Adaptation, Vision, Audition, Multimodal, Crossmodal, Discrimination, Action, Perception

## Abstract

Prolonged exposure, or adaptation, to a stimulus in 1 modality can bias, but also enhance, perception of a subsequent stimulus presented within the same modality. However, recent research has also found that adaptation in 1 modality can bias perception in another modality. Here, we show a novel crossmodal adaptation effect, where adaptation to a visual stimulus enhances subsequent auditory perception. We found that when compared to no adaptation, prior adaptation to visual, auditory, or audiovisual hand actions enhanced discrimination between 2 subsequently presented hand action sounds. Discrimination was most enhanced when the visual action “matched” the auditory action. In addition, prior adaptation to a visual, auditory, or audiovisual action caused subsequent ambiguous action sounds to be perceived as less like the adaptor. In contrast, these crossmodal action aftereffects were not generated by adaptation to the names of actions. Enhanced crossmodal discrimination and crossmodal perceptual aftereffects may result from separate mechanisms operating in audiovisual action sensitive neurons within perceptual systems. Adaptation-induced crossmodal enhancements cannot be explained by postperceptual responses or decisions. More generally, these results together indicate that adaptation is a ubiquitous mechanism for optimizing perceptual processing of multisensory stimuli.

**Electronic supplementary material:**

The online version of this article (doi:10.3758/s13414-016-1199-z) contains supplementary material, which is available to authorized users.

Prolonged exposure, or adaptation, to visual and auditory stimuli, often results in “aftereffects,” where perception of subsequent test stimuli is biased away from the adapted stimulus. These aftereffects can result from adaptation to simple (Gibson & Radner, 1937) and complex (Barraclough, Keith, Xiao, Oram, & Perrett, 2009) visual stimuli, and simple (Grantham, 1989) and complex (Zaske, Schweinberger, & Kawahara, 2010) auditory stimuli. Adaptation is thought to result in a temporary suppression of the firing rate of neurons coding the adapting stimulus (i.e., the stimuli to which they are “tuned”). Those neurons that selectively respond to other stimuli are little, or not affected by this adaptation to their nonpreferred stimuli. This stimulus-specific reduction in cell responses following adaptation has been seen at multiple levels in perceptual processing, including in early (e.g., V1; Saul & Cynader, 1989) and later (e.g., the superior temporal sulcus, STS; Baylis & Rolls, 1987) visual processing areas, as well as in early auditory processing (e.g., ganglion cells; Yates, Robertson, & Johnstone, 1985).The use of fMRI-adaptation in humans suggests suppression of auditory cells coding more complex stimuli in later stages of auditory processing (e.g., STS; Belin & Zatorre, 2003). These adaptation-induced reductions in firing rates of discrete groups of neurons are thought to play a role in the aftereffects observed in human perception (Clifford, Wenderoth, & Spehar, 2000; Kohn, 2007).

Adaptation, however, can also selectively enhance perception around the adapting stimulus. For example, adapting to untrustworthy faces results in improvements in the ability to discriminate between untrustworthy faces while having little effect on the perception of trustworthy faces (Keefe, Dzhelyova, Perrett, & Barraclough, 2013). Improved perceptual discrimination around the adaptor has also been seen following adaptation to motion (Phinney, Bowd, & Patterson, 1997), speed (Clifford & Langley, 1996), face viewpoint (Chen, Yang, Wang, & Fang, 2010), face gender (Yang, Shen, Chen, & Fang, 2011), and face race (Rhodes, Watson, Jeffery, & Clifford, 2010), although some studies of improvements in face perception following adaptation have been more equivocal (e.g., Ng, Boynton, & Fine, 2008; Rhodes, Maloney, Turner, & Ewing, 2007). Thus, at the expense of absolute sensitivity, adaptation can increase differential sensitivity, enabling the observer to detect smaller differences around the adapted stimulus. At the single-cell level, adaptation can result in the narrowing of the tuning functions of single units (e.g., Kohn & Movshon, 2004), thereby maximizing the effective neural bandwidth for the representation of subsequent stimuli. Adaptation, therefore, can have a functional benefit to the observer, optimizing the limited dynamic range of perceptual pathways for the coding of future stimuli (Clifford et al., 2000).

Several studies have indicated that adaptation in one modality can bias perception in another modality (e.g., Hills, Elward, & Lewis, 2010; Kitagawa & Ichihara, 2002; Konkle, Wang, Hayward, & Moore, 2009; Pye & Bestelmeyer, 2015; Skuk & Schweinberger, 2013; Zaske et al., 2010). In Hills et al. (2010), for example, adaptation to the voice of a familiar individual resulted in a reduction in the likelihood of subsequently presented morphed faces being categorized as the same identity as the voice. Often, evidence of crossmodal aftereffects has been used to argue for the existence of supramodal representations of simple stimuli (e.g., motion; Konkle et al., 2009), as well as more complex social information (e.g., emotion; Pye & Bestelmeyer, 2015). In all these previous studies, however, evidence for crossmodal adaptation has relied upon measurements of shifts in the central tendency of psychometric functions fitted to observers’ estimates of single stimuli (Hills et al., 2010; Pye & Bestelmeyer, 2015), or adaptation-induced biases in the categorization of individually presented ambiguous stimuli (Kitagawa & Ichihara, 2002; Konkle et al., 2009; Skuk & Schweinberger, 2013; Zaske et al., 2010). In these experiments, on each trial, following adaptation to a stimulus presented in one modality, a single test stimulus in another modality was presented, and observers were required to classify the test stimulus as belonging to one of up to three categories. The choice of category to which the observer might assign the stimulus depends not only upon the observer’s sensory evidence but also their criteria for assigning categories to the sensory evidence (Green & Swets, 1974); therefore, adaptation-induced changes in observers’ perception are indistinguishable from changes in their criteria. Although, these previous studies have reported crossmodal aftereffects as perceptual biases due to neural adaptation, the use of the “method of single stimuli” in all of these studies precludes their ability to distinguish genuine adaptation-induced perceptual biases from postperceptual response biases (see Morgan, Dillenburger, Raphael, & Solomon, 2012; Morgan, Melmoth, & Solomon, 2013; Storrs, 2015).

In this paper, and in contrast to previous studies, we wanted to assess whether adaptation in one modality could enhance the discrimination of stimuli presented in another modality, and to determine whether crossmodal adaptation resulting from perceptual processes could be delineated from post-perceptual processes. We adapted a design to measure adaptation-induced changes in the discrimination of two test stimuli (cf. Keefe et al., 2013), however, in this study we measured the effect of adaptation to a stimulus presented in one modality on the discriminability of two stimuli presented in another modality. By employing this approach, we can also be certain we are measuring perceptual effects rather than post-perceptual response biases (Morgan et al., 2013; Storrs, 2015). In Experiment 1, we tested the effect of adapting to visual, auditory, and audiovisual hand actions on the discrimination of subsequent hand action sounds. We choose these stimuli because previous research shows that visual adaptation to hand actions results in biases in recognition of subsequent visual hand actions (Barraclough et al., 2009; de la Rosa, Streuber, Giese, Bulthoff, & Curio, 2014). In addition, many hand actions are typically multimodal in nature, and the integration of visual and auditory information can help us interpret and understand actions better (Arrighi, Marini, & Burr, 2009; Petrini, Russell, & Pollick, 2009; Schutz & Lipscomb, 2007; Thomas & Shiffrar, 2010; van der Zwan et al., 2009). Based upon the prior findings that adaptation in one modality can influence perception in another modality, we predicted that adaptation to auditory, visual, and audiovisual stimuli would enhance the ability of observers to discriminate the action sound. Furthermore, given that when sight and sound are presented together perception is typically enhanced (e.g., Fort, Delpuech, Pernier, & Giard, 2002; Giard & Peronnet, 1999), and that audiovisual adaptors can generate larger aftereffects than unimodal adaptors (Kitagawa & Ichihara, 2002), we also predicted that discrimination would be more enhanced by the audiovisual adaptor compared to either of the unimodal adaptors. In Experiment 2, we replicated our test of visual adaptation on the ability of observers to discriminate action sounds while assessing if crossmodal adaptation was dependent upon the hand action used as the adapting stimulus. Adaptation-induced perceptual enhancement depends upon similarity between the adapting and test stimuli (Kohn, 2007); we predicted, therefore, that when the adapting action was the same as the test actions, enhancement in discrimination would be greater than when the adapting action did not match the test action.

In two final experiments, we tested whether the adapting hand action stimuli used in the first two experiments could induce the more commonly observed biases in perception of subsequent test stimuli (aftereffects). Although we used methodology similar to that critiqued above (and therefore subject to similar criticisms), we wanted to evaluate whether our hand-action stimuli could induce both crossmodal biases in perception and crossmodal enhancements of perception. In Experiment 3, we tested the effect of adapting hand actions on the perception of subsequent ambiguous hand-action sounds. We predicted that adaptation to one action would make ambiguous test sounds appear less like the adapting action (cf. Barraclough & Jellema, 2011; Barraclough et al., 2009). Furthermore, similar to the design used by Kitagawa and Ichihara (2002) to measure crossmodal aftereffects with more simple stimuli, we tested the magnitude of aftereffects generated by auditory, visual, and two different audiovisual adaptors: one where the visual action was congruent with the action sound and one where the visual action was incongruent with the action sound. As for previous demonstrations of crossmodal (e.g., Kitagawa & Ichihara, 2002; Skuk & Schweinberger, 2013) aftereffects with different stimuli, we predicted that aftereffects would result from adaptation to auditory, visual, and audiovisual adaptors. Finally, in Experiment 4, we conducted an additional experiment in part to replicate our test of the effect of visual adaptation on auditory sound perception measured in Experiment 3, but also to determine if the visually induced auditory aftereffects were based upon the perceptual characteristics of the visual stimulus or were due to a more general action concept adaptation. Following Schweinberger et al. (2008), we additionally tested whether adaptation to the name of the action induced auditory action aftereffects.

## General method

### Participants

Participants were staff or students from the University of York or the University of Hull. All had normal or corrected-to-normal vision. Participants gave informed consent and either received course credit or were paid for their participation. Experiments were approved by the ethics committees of the Departments of Psychology, University of York and University of Hull, and were performed in accordance with the ethical standards laid down in the 1990 Declaration of Helsinki. We aimed to recruit approximately 20 participants, through opportunity sample, for each study based upon similar sample sizes used in previous experiments (e.g., Barraclough, Ingham, & Page, 2012; Barraclough & Jellema, 2011); variance in the number of participants in each study was due to the different numbers signing up to take part in experiments during the period of each study. All participants were naïve to the aims of the study, except in Experiments 1 and 2, where two of authors were participants (B. D. K. & N. E. B.), and in Experiment 3 where one of the authors was a participant (S. P.).

### Stimuli

A male hand knocking (fist closed) and slapping (fist open) on a heavy wooden desk were filmed using a Canon XL1s digital camcorder (720 × 576 pixels, 25 fps), while action sounds were simultaneously recorded in stereo (16 bit, 48 kHz) using an externally connected microphone (Sennheiser K6 ME66). Audiovisual film footage was edited using Adobe Premier Pro 5.0 to generate 680-ms (17 frame) movie clips of each action. Each movie was edited such that the hand made contact with the wood at 200 ms (Frame 6). Each frame of each action movie was converted to grayscale, and luminance equalized across all frames (MATLAB; The MathWorks, Natick, MA).

**Fig. 1:**
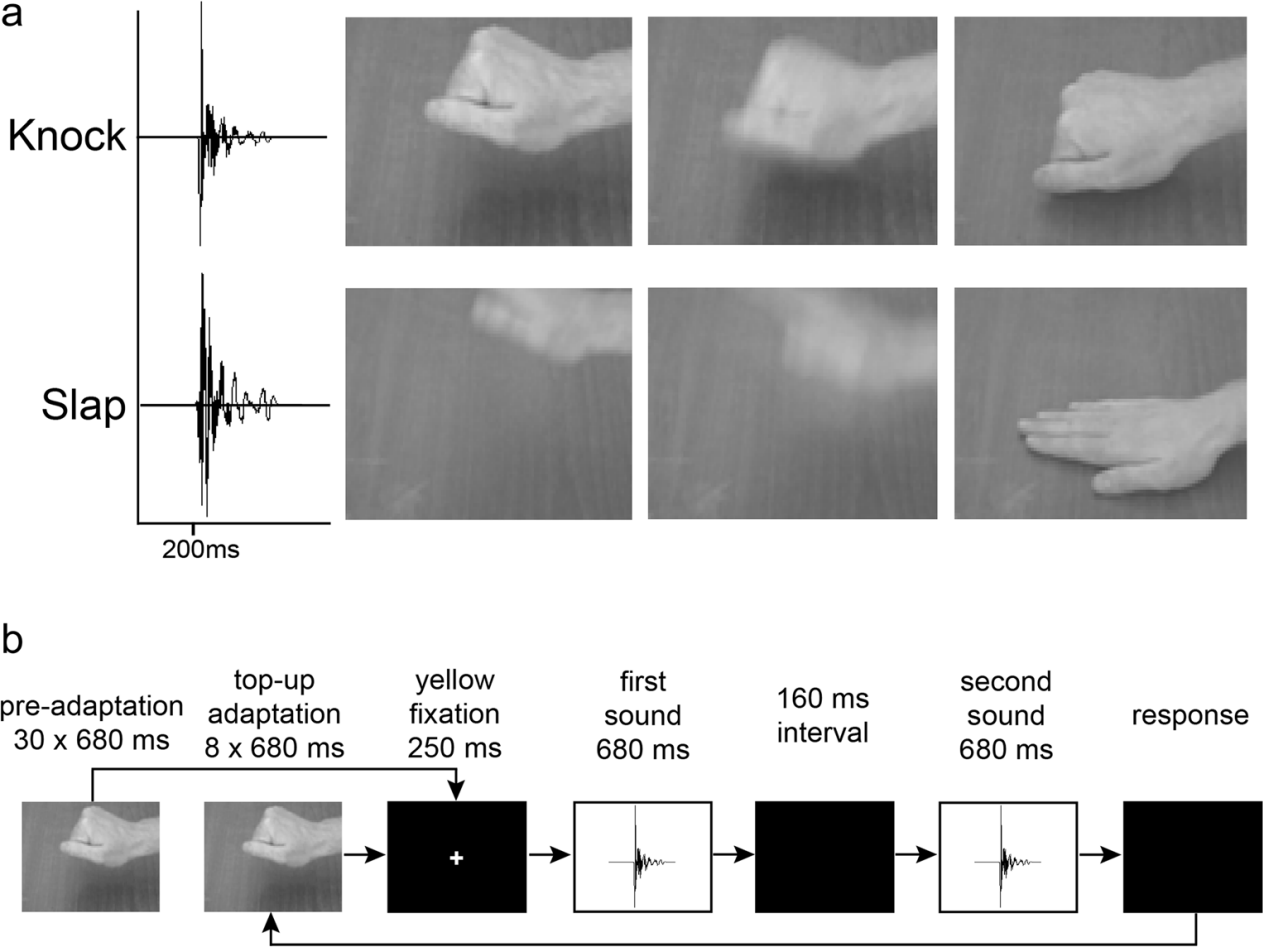
Stimuli and experimental procedure for Experiment 1. (**a**) Waveforms show audible component of knock and slap action sounds. *X*-axis shows stimulus duration of 680 ms and sound onset at 200 ms. Images illustrate grayscale versions of individual frames (left to right: 2, 4, 7) from each visual action. (**b**) Schematic description of the experimental procedure in Experiment 1 for the V adapting condition. Following preadaptation on the first trial and top-up adaptation on subsequent trials to visual knocks, two slightly different knock action sounds (the standard and the comparison) were presented sequentially. Participants were required to indicate which sound appeared most like a knock

The auditory signals from the original film were subsequently resampled at 16-bit/44.1 kHz. Although the duration of each action-sound file was 680 ms, the audible component of each sound commenced 200 ms into the file. Extremely low frequency components, below 100 Hz, were removed using a high-pass filter, and then each found file was equalized so that they were presented at 64 dB intensity (Praat; http://www.fon.hum.uva.nl/praat/). The auditory stimuli and example frames from both audiovisual actions are illustrated in Fig. 1a.

Test stimuli were ambiguous action sounds in between the knock and slap action sounds. Because hand action sounds do not contain a recognizable fundamental frequency, it was not possible to morph between them, as might be performed with vocal stimuli (e.g., Moulines & Charpentier, 1990; Specht, Rimol, Reul, & Hugdahl, 2005). We therefore generated action-sound blends (Sony Sound Forge 10 Pro; http://www.sonycreativesoftware.com/soundforge) between the resampled and filtered knock action sound and the slap action sound by adding together knock and slap sounds so that each sound contributed a fixed percentage of the amplitude of the final stimulus. Through this process it was possible to generate blended actions sounds in percentage steps ranging from 1 %slap/99 %knock (see Supplementary Material, Sound File 1) through to 99 %slap/1 %knock (Supplementary material Sound File 2).

The blended auditory stimuli were then imported back into Adobe Premier to generate different adapting stimuli. Adapting stimuli used in Experiments 1 included the (predominantly; 20 %slap/80 %knock) knock sound presented alone (A), the (20 %slap/80 %knock) knock sound presented simultaneously with the congruent visual knock (AVc), or the visual knock presented alone (V). In Experiment 2, adapting stimuli included the visual knock presented alone (Vknock) and visual slap action presented alone (Vslap).

For Experiments 3 and 4, adapting stimuli were slightly different, and stimuli for both action types were generated. These included the action sound presented alone (A), the action sound presented simultaneously with the congruent visual information (AVc), the action sound presented simultaneously with the incongruent visual information (AVi), or the visual action presented alone (V). Incongruent adapting stimuli (AVi) were comprised of one auditory action (e.g., the knock sound) paired with the other visual action (e.g., slap visual movie). During Experiments 3 and 4, auditory components in the adapting stimuli were such that the relevant action was maximal (knock sounds: 0 %slap/100 %knock; slap sounds: 100 %slap/0 %knock). For audiovisual (AVc, AVi) stimuli referred to in this study we reference the action conveyed by the auditory component in all cases—for example, the knock AVi stimulus would contains an auditory knock component and a visual slap component.

Auditory adapting stimuli were made by rendering out from Adobe Premier the auditory component of the two actions as separate .wav files. Visual adapting stimuli were made by rendering the visual component of the two actions as separate .avi files. For all audiovisual adapting stimuli, the auditory and the visual components of the actions were synchronous. This was achieved by aligning the 680-ms auditory file with the appropriate 680-ms visual file within Adobe Premier, and then rendering the combined data as an .avi file.

## Experiment 1

In the first experiment, we tested if the perception of action sounds was enhanced by prior adaptation to actions presented in different modalities. Eighteen participants took part (13 female, mean age = 25.44 years, *SD* = 5.48).

### Method

#### Experimental procedure

A PC running MATLAB 2010a and the Psychophysics Toolbox was used to control the experiment, present the stimuli, and record participant responses. Participants sat in a dimly lit soundproofed booth .6 m away from a 24-in. TFT monitor (Acer GD245HQ, 1920 × 1080 pixels, 100-Hz refresh rate) on which all visual stimuli were presented. Visual stimuli were presented on a black background and subtended approximately 22.3° × 16.6° at the eye. Participants wore Sennheiser HD280 Pro headphones, from which all auditory stimuli were presented.

We measured action sound discrimination thresholds (just noticeable differences: JNDs) using a 2-AFC procedure when no adaptor was present, and following presentation of different adaptors. Comparing discrimination thresholds between the no-adaptation condition and the adaptation conditions gives a measure of the effect of adaptation on perceptual discrimination. This methodology has been used extensively to measure the effect of adaptation on visual discrimination thresholds (e.g., Abbonizio, Langley, & Clifford, 2002; Chen et al., 2010; Clifford, Wyatt, Arnold, Smith, & Wenderoth, 2001; Keefe et al., 2013; Phinney et al., 1997; Regan & Beverely, 1985; Yang et al., 2011). Just noticeable differences were measured for a (predominantly) knock action sound (20 %slap/80 %knock; see Supplementary Material, Sound File 3) under four conditions: following no adaptation and following AVc, V, and A adaptors. In conditions where the adapting stimulus contained an auditory component, the knock sound adaptor (20 %slap/80 %knock) was always *identical* to the “standard” test sound (see below). The four different adapting conditions were tested in separate blocks, on separate days, to reduce the possibility of adaptation that was generated during one block of testing influencing perception in a subsequent block of testing. Block occurrence was counterbalanced across participants. During blocks of testing with adaptors, there was first a 20.4-s period of preadaptation where the adapting knock action was repeated 30 times followed by a 250-ms interval during which a yellow fixation cross appeared in the center of the screen. Following the interval, two test sounds (a standard sound and a comparison sound) were presented 160 ms apart. Participants indicated with a key press which of the two sounds, first or second, was most like a knock action sound. On the following trials, there were first eight repeats of the adapting action, a 250-ms interval with the centrally presented yellow fixation cross, and then the two test stimuli separated by 160 ms (see Fig. 1b). For the no-adaptation condition, there was no preadaptation presentation of adapting stimuli; participants were first presented with a blank screen with a centrally presented yellow fixation cross for 250 ms, and then the two test stimuli, separated by 160 ms.

On every trial, participants had 2 s to respond to the test stimuli; if a response was not recorded during this period, the trial was immediately repeated. Once a response was registered, there was a 500-ms interval before the next trial. The standard test sound always consisted of a 20 %slap/80 % knock action sound, while the degree of knock action contributing to the comparison test sound varied using adaptive staircase procedures. The order of the standard and comparison within each trial was randomized. Participants completed each of the four conditions with each of four interleaved staircase reversal rules (1 up, 2 down; 2 up, 1 down, 1 up, 3 down; 3 up, 1 down). We did not determine thresholds from the staircase endpoints; these procedures were used to distribute trials at informative points along the psychometric function (Levitt, 1971), which was fitted using the data from all the trials. Staircase step sizes were initially 8 % and were halved on each of the first three reversals. The staircase quit after 14 reversals, typically resulting in ~45 trials per staircase type (~180 trials per psychometric function).

Perceptual learning can produce marked increases in performance over the short term, resulting in strong order effects (Poggio, Fahle, & Edelman, 1992). To mitigate these order effects, participants first practiced the discrimination task until their performance plateaued. Before we tested each participant in the experiment, we assessed their ability to discriminate the action sounds using exactly the same procedure as the no-adaptation block of testing. These practice blocks were repeated until the participants showed no improvement in their ability to discriminate the action sounds. This was determined by fitting psychometric functions to the data obtained from each block, and once the JND calculated from the data in block *n* was less than 1.5 standard deviation from the JND calculated from the data in block *n* - 1, then these practice sessions were stopped, and the participant moved on to start the experiment. The performance of two participants declined with practice, and therefore they did not attempt the experiment.

#### Analysis

For each participant and condition, JNDs were computed by first fitting cumulative Gaussian psychometric functions to the data using a maximum likelihood method of fit in MATLAB, while allowing the central tendency (mu) and the standard deviation (sigma) to freely vary. We divided the resulting standard deviations by $\surd 2$ to give an estimate of the standard deviation on a single interval (because we used a 2-IFC procedure; Green & Swets, 1974). The resulting values are JNDs because they indicate the percentage change in the action sound that can be discriminated at the ~76 % level. We tested the analysis of the influences of different forms of adaptation over the no-adaptation condition with planned comparisons based upon our original hypotheses that adaptation (of any kind) would enhance discrimination. In addition to post hoc *t* tests to evaluate significant differences between the unimodal and multimodal adaptors, we used Bayesian *t* tests (Dienes, 2008, 2011) to quantify the evidence in favor for or against a beneficial influence of having multimodal adaptation over unimodal adaptation. The resulting Bayes factor (B) quantifies how much more (or less) likely the data are under the alternative hypothesis than under the null hypothesis. For example, B(AV < A) = 3.0 would indicate that the data are 3 times more likely to indicate that audiovisual adaptation effects are greater than effects to visual adaptation alone compared to the null hypothesis, whereas B(AV < A) = 1/3 would indicate that the data are 3 times more likely to indicate the null hypothesis over the alternative that audiovisual adaptation effects are greater than effects to visual adaptation alone. The suggested convention (Jeffreys, 1961) is that Bayes factors above 3 indicate substantial evidence for the alternative hypothesis (audiovisual adaptation effects are substantially greater than unimodal adaptation effects), Bayes factors below 1/3 indicate substantial evidence for the null hypothesis (audiovisual adaptation effects are similar to unimodal effects), while values in between 3 and 1/3 indicate neither support for the alternative nor the null hypothesis.

### Results

**Fig. 2:**
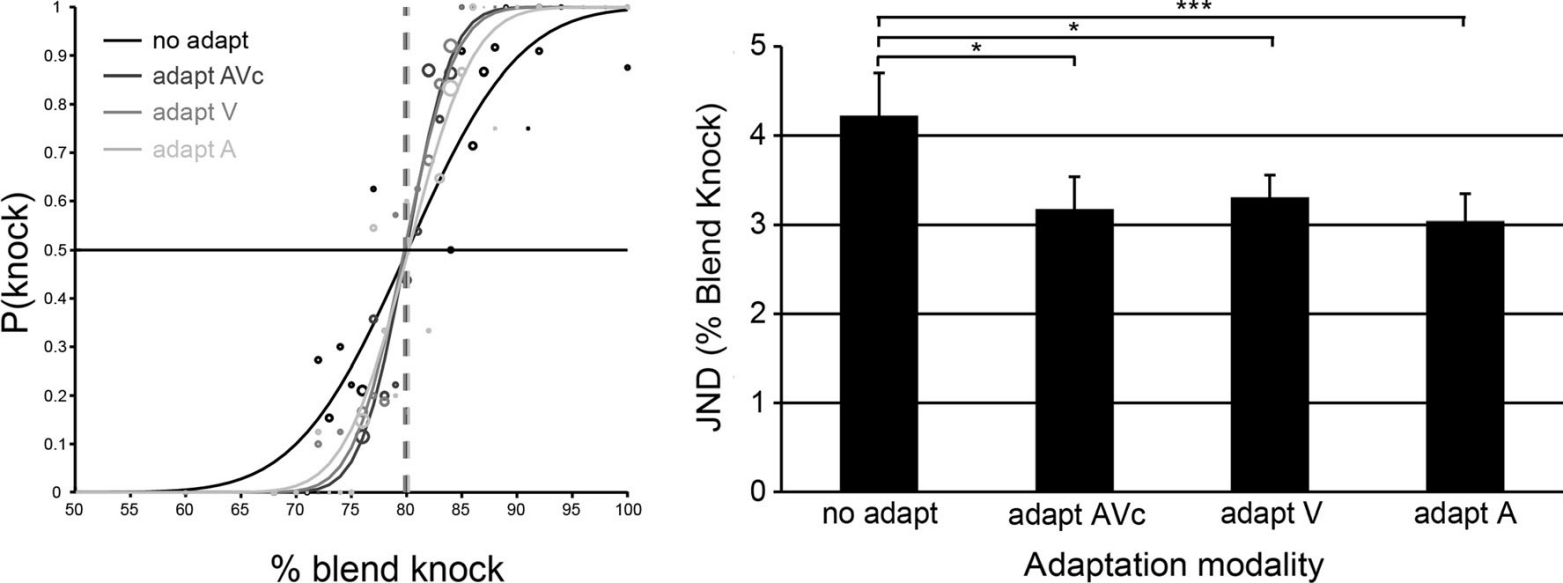
Adaptation improves action sound discrimination. Left panel: psychometric functions fitted to the data of an example individual under no adapt (*black*), AVc (*dark gray*), V (*mid-gray*) and A (*light gray*) conditions. The *circles* illustrate data points, where circle magnitude corresponds to the number of trials for that data point. Cumulative Gaussian functions are fitted to the data, the slopes of which are used to derive JNDs; these show that adaptation increases the ability of this individual to discriminate the knock action sounds. *Right panel*: average JNDs across all participants tested. *Error bars* denote ±*SEM*. Asterisks denote a significant difference between conditions based upon planned contrasts. ***p < .005. *p < .05

Adaptation had a significant influence on the ability to discriminate the perception of knock sounds (see Fig. 2) *F*(2.13, 36.23) = 5.54, *p* = .007, η_p_^2^ = .25, 95 % CIs no adapt [3.22, 5.23], adapt audiovisual [2.46, 3.94], adapt visual [2.78, 3.83], adapt auditory [2.39, 3.69], Greenhouse–Geisser correction applied. Auditory, V and AVc adaptors all increased the ability of participants to discriminate action sounds (all planned contrasts), *F*s(1, 17) > 5.25, *p*s < .035, η_p_^2^s > .24. This effect of adaptation was reflected in a decrease in the standard deviation, or steepening of the slopes, of the fitted cumulative Gaussian functions (illustrated in Fig. 2). JNDs did not differ significantly following adaptation to visual, auditory or audiovisual adaptors, AV (*M* = 3.17, *SD* = 1.54) versus V (*M* = 3.31, *SD* = 1.06), *t*(17) = −.52, *p* = .61, Cohen’s *d* = −.17, *r* = −.08, CI [−.67, .41]; AV (*M* = 3.17, *SD* = 1.54) versus A (*M* = 3.04, *SD* = 1.30), *t*(17) = .45, *p* = .66, Cohen’s *d* = −.13, *r* = −.06, CI [−.50, .77]; V (*M* = 3.31, *SD* = 1.06) versus A (*M* = 3.04, *SD* = 1.30), *t*(17) = 1.24, *p* = .23, Cohen’s *d* = .23, *r* = .11, CI [−.19, .72]. Bayes factors calculated for the comparison between audiovisual and unimodal adaptors (B[AV < V] = 0.07; B[(AV < A] = 0.18) provided evidence for the null hypothesis, that is, adaptation to an audiovisual adaptor did not have a beneficial influence on auditory discrimination compared to unimodal adaptors.

## Experiment 2

Experiment 2 was conducted to replicate the crossmodal enhancement observed previously, and to determine if adaptation depended upon the action used as the adapting stimulus. Adaptation-induced perceptual enhancement is dependent upon similarity between the adapting and test stimuli (Kohn, 2007); we therefore tested if visual knock actions enhanced knock sound discrimination more than visual slap actions. Twenty-two participants took part in Experiment 2 (17 female, mean age = 24.30 years, *SD* = 5.34).

### Method

#### Experimental procedure

We measured JNDs for knock action sounds (20 %slap/80 %knock) under three conditions: following no adaptation, following adaptation to a visual knock alone (Vknock), and following adaptation to a visual slap alone (Vslap). Otherwise, the experimental procedure was identical to that used in Experiment 1.

#### Analysis

For each participant and condition, JNDs were computed as for Experiment 1. Similar to our analysis for Experiment 1, we supplemented a conventional paired-samples *t* test with a Bayesian *t* test to quantify the evidence for the knock adaptor enhancing discrimination over that of the effect of the slap adaptor.

### Results

**Fig. 3:**
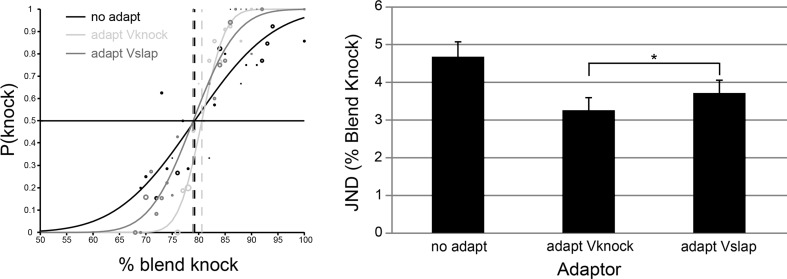
Action type influences action sound discrimination. *Left panel*: psychometric functions fitted to the data for an example individual under no adapt (*black*), adapt Vknock (*light gray*) and adapt Vslap (*dark gray*) conditions. The *circles* illustrate data points, where circle magnitude corresponds to the number of trials for that data point. Cumulative Gaussian functions are fitted to the data points, the slopes of which are used to derive JNDs and show that adaptation increases the ability of this individual to discriminate the knock action sounds. *Right panel*: average JNDs across all participants tested. *Error bars* denote ±*SEM. Asterisks* denote a significant difference between the Vknock and Vslap conditions. *p < .05

As for Experiment 1, visual adaptation resulted in significant changes in the ability to discriminate different action sounds (see Fig. 3), *F*(1.2, 25.2) = 8.07, *p* < .006, η_p_^2^ = 0.28, 95 % CIs no adapt [3.88, 5.55], adapt Vknock [2.59, 3.98], adapt Vslap [3.03, 4.45], Greenhouse–Geisser correction applied. The effect of adapting to the sight of knock actions enhanced discrimination of knock sounds significantly more than the effect of adapting to the sight of slap actions, Vknock (*M* = 3.28, *SD* = 1.57) versus Vslap (*M* = 3.74, *SD* = 1.60), *t*(21) = −2.33, *p* = .030, Cohen’s *d* = −.29, *r* = −.14, CI [−.87, −.05], B(Vknock < Vslap) = .1.98, whereas the Bayes factor indicates that there is more evidence for the hypothesis that adaptation to knock actions enhances knock discrimination more than adaptation to slap actions when compared with the null hypothesis; this evidence, however, is not substantial.

## Experiment 3

Experiment 3 tested if adaptation to the hand actions used in Experiments 1 and 2 could, in addition, generate more typical biases in perception (aftereffects; cf. Barraclough et al., 2009). Seventeen participants took part (12 female, mean age = 22.74 years, *SD* = 1.85).

### Method

#### Experimental procedure

A PC running MATLAB 2006a and the Cogent Toolbox was used to control the experiment, present the stimuli, and record participant responses. Participants sat in a sound-attenuated booth, approximately .6 m from a flat-screen 22-in. CRT monitor (Philips 202P40, 1280 × 1024 pixels, 100-Hz refresh rate) and wore Sennheiser HD280 Pro headphones. All visual stimuli were presented on the screen and all auditory stimuli though the headphones. Visual action movies were shown in the middle of a mid-gray (luminance = 9.7 cd m^−1^) background at full resolution (720 × 576 pixels) and subtended approximately 22.3° × 16.6° at the eye. This was achieved by rendering on screen in sequence each frame from the action movie as a bitmap at a speed of 25 frames/second.

We tested the effect of adapting to different modality actions on the perception of subsequent action sounds. Participants adapted to knock and slap actions presented in the A, AVc, AVi, and V modalities while they indicated their perception of subsequently presented ambiguous action sounds (30 %slap/70 %knock; 40 %slap/60 %knock; 50 %slap/50 %knock; 60 %slap/40 %knock; 70 %slap/30 %knock; 80 %slap/20 %knock; see Supplementary Materials, Sound Files 4–9). The additional 80 % slap sound was included as a test sound to ensure that the range of actions broadly extended over the midpoint between the slap and knock sounds. Prior pilot testing had indicated that the slap sound was slightly less recognizable, and the point of subjective equivalence between the slap and knock sounds lay closer to the slap action than the physical midpoint between the action sounds.

Each adapting condition (two actions × four modalities) was tested in eight separate blocks of testing. Block occurrence was counterbalanced across participants. Each block of testing consisted of a preadaptation test phase, an adaptation phase, and a postadaptation test phase. In the pre- and postadaptation test phases, test action sounds were presented alone with no adaptation. Both pre- and postadaptation testing were performed to explore whether judgments of action sounds drifted over time due to the intervening adaptation phase. For analysis, these were combined to calculate a baseline measure of how each participant judged the action sounds when presented without adapting stimuli for that block of testing. In each of the pre- and postadaptation phases, the six test sounds were presented eight times each, in a pseudorandom order. On each trial, a yellow fixation cross appeared on screen for 200 ms in advance of the test sound played through the headphones. Following sound presentation, participants had to indicate whether the sound was of a knock action or a slap action by pressing one of two keys on a keyboard. Following a period of 500 ms after registering a key press, the computer advanced to the next trial.

Between the pre- and postadaptation phases was the adaptation phase of testing. At the start of this phase, the adapting action was presented repeatedly 60 times (duration = 40.8 s). Following this initial adaptation, a “top-up” adaptation of five repeats (duration = 3.4 s) was delivered, followed by a brief interstimulus interval (ISI) of 200 ms, during which a yellow fixation cross appeared on the center of the screen, and then the test sound was presented. As for the pre- and postadaptation phases, participants had to indicate whether the sound was of a knock action or a slap action. Following a period of 500 ms after registering a key press, the computer advanced to the next trial. During the adaptation phase, the six test sounds were presented eight times each, in a pseudorandom order.

#### Analysis

We scored all participant responses on a 0 to 1 scale, where 0 indicated a slap response and 1 indicated a knock response. For each participant, we calculated the mean score separately for each of the adaptation conditions (two actions × four modalities), as well as for the pre- and postadaptation phases for each condition separately. Mean scores for test stimuli were compared between each pair of pre- and postadaptation phases to assess if the adaptation phase had generated a longer term shift in the action sound ratings. In addition, scores were averaged across each pair of pre- and postadaptation phases to generate a condition specific control to which the effect of each adapting stimulus could be compared. Aftereffects for slap and knock actions and the four modalities were calculated by subtracting the mean scores during the specific control from the mean scores during the adaptation phase. Aftereffects could vary between +1 and −1; positive aftereffects indicated that, compared to the control, test sounds appeared more like a knock, negative aftereffects indicated that, compared to the control, test sounds appeared more like a slap. For adaptors presented in each modality, Bayes factors were calculated to quantify the evidence for the presence of aftereffects over the null hypothesis.

### Results

**Fig. 4:**
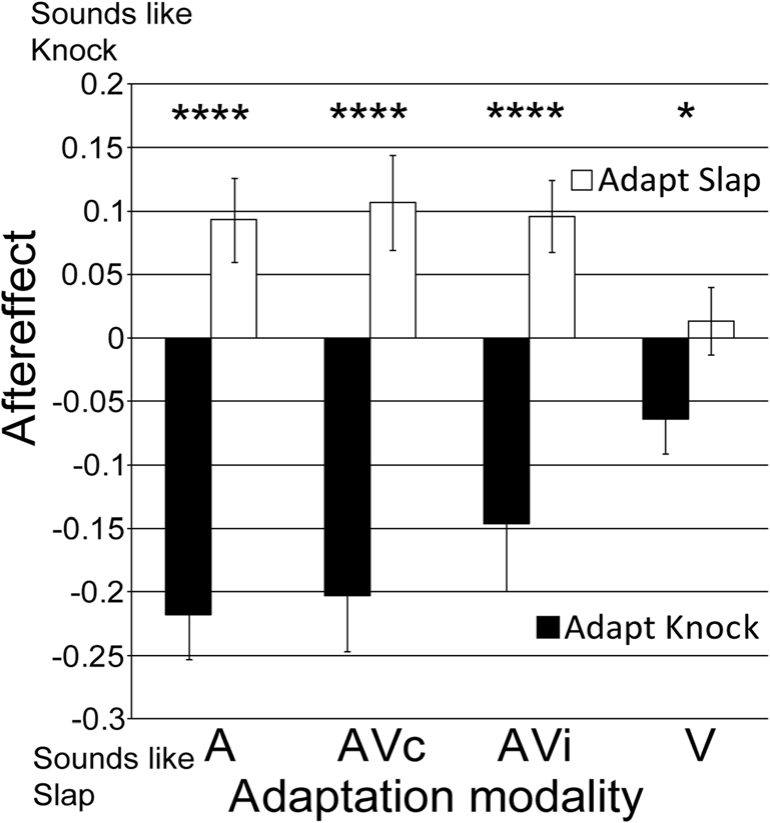
Auditory aftereffects generated by unimodal and multimodal adaptation. Ambiguous action sound perception following adaptation to knocks (*dark bars*) and slaps (*white bars*). Adapting stimuli were presented as auditory stimuli alone (A), as congruent audiovisual stimuli (AVc) where the visual stimulus matched the sound, as incongruent audiovisual stimuli (AVi) where the visual action was incongruent to the sound, and as visual stimuli alone (V). *Error bars* denote ±*SEM. Asterisks* denote a significant difference between action conditions for each adaptor modality. ****p < .001. *p < .05

Preadaptation and post-adaptation measures of participants’ judgments of the action sounds were not significantly different from each other, pretest (*M* = .51, *SD* = .15) versus posttest (*M* = .51, *SD* = .12), *t*(16) = .14, *p* > .250, Cohen’s *d* = .02, *r* = .01, 95 % CI [−.04, .05], B(pretest > posttest) = .03, and were averaged to generate a baseline to which the effects of adaptation could be compared. The adapting action had a significant effect on the perception of action sounds (see Fig. 4), *F*(1, 16) = 41.14, *p* < .001, η_p_^2^ = 0.72, 95 % CIs adapt knock [−.23, −.09], adapt slap [.02, .14]. Prior adaptation to knock actions made the subsequently presented test action sounds appear more like slap sounds. While adapting to slap actions had the opposite effect, here, test action sounds appeared more like knock sounds. The modality of the adaptor also had a significant influence on the magnitude of the aftereffects (Adapting Action × Modality Interaction): *F*(3, 48) = 9.09, *p* < .001, η_p_^2^ = 0.36, CIs adapt knock A [−.29, −.14], AVc [−.29, −.11], AVi [−.26, −.03], V [−.12, −.01], adapt slap A [.02, .16], AVc [.03, .19], AVi [.03, .16], V [−.04, .07]. There was no main effect of modality, *F*(3, 48) = 1.40, *p* > .250, η_p_^2^ = 0.08, CIs A [−.12, .00], AVc [.11, .01], AVi [−.09, .04], V [−.07, .02]. Irrespective of the modality of the adaptor, all aftereffects were significantly different from each other, whereas auditory only (A) aftereffects were largest (*M* = .311, *SD* = .17), *t*(16) = 7.55, *p* < .001, Cohen’s *d* = 2.22, *r* = .74, CI [.22, .40], B > 1,000, congruent audiovisual (AVc) next largest (*M* = .31, *SD* = .23) *t*(16) = 5.55, *p* < .001, Cohen’s *d* = 1.84, *r* = .76, CI [.19, .43], B > 1,000, incongruent audiovisual (AVi) next largest (*M* = .24, *SD* = .25), *t*(16) = 4.00, *p* < .001, Cohen’s *d* = 1.37, *r* = .56, CI [.11, .37], B = 731, and visual (V) aftereffects smallest but still significant (*M* = .07, *SD* = .11), *t*(16) = 2.70, *p* = .016, Cohen’s *d* = .69, *r* = .33, CI [.02, .07], B = 6.76. Bayes factors indicated evidence for auditory aftereffects generated by all modality adaptors.

## Experiment 4

We conducted an additional experiment in order to replicate our finding that visual adaptation generates auditory aftereffects; in addition to determine if the visually induced auditory aftereffect was based upon the perceptual characteristics of the visual stimulus or was due to a more general action concept adaptation. Following Schweinberger et al. (2008), we tested whether adaptation to the name of the action induced auditory action aftereffects. Twenty participants took part (12 female, mean age = 22.61 years, *SD* = 5.26).

### Method

#### Experimental procedure

This experiment was very similar to Experiment 3, with the same methods of presentation, the same test stimuli, and the same experimental procedure. Adapting stimuli were knock and slap actions presented in the AVc and V modalities, as well as an orthographic modality (O). For the orthographic modality, the words *slap* and *knock* in black Arial font, with a width of 18°, were presented on the gray background in exactly the same way as for the visual actions. We hypothesized that if the adaptation effects were conceptual in nature, then presentation of action words would induce significant auditory aftereffects similar to those induced by visual adaptation to the action movies.

#### Analysis

For each participant and condition, aftereffects were computed as for Experiment 3. For adaptors presented in each modality, Bayes factors were calculated to quantify the evidence for presence of aftereffects over the null hypothesis.

### Results

Preadaptation (*M* = .55, *SD* = .11) and postadaptation (*M* = .47, *SD* = .08) measures of participants’ judgments of the action sounds were significantly different from each other, *t*(19) = 2.29, *p* < .034, Cohen’s *d* = .82, *r* = .38, 95 % CI [.00, .15], B(pretest > posttest) = 1.16. In the preadaptation test, participants judged the ambiguous test sounds to appear more like a knock action (*M* = 0.55, *SD* = 0.11) than in the postadaptation test (*M* = 0.47, *SD* = 0.08), where the test sounds appeared more like a slap action. The Bayes factor indicated that there was neither evidence for, nor against, the hypothesis that the baseline judgments would be the same during pre- and posttesting. The intervening adapting stimuli were counterbalanced such that knock and slap actions occurred with equal frequency and an equal number of times at each position in the testing sequence, and so this represents a general trend in this group of participants to shift their internal criterion towards slap actions over time, rather than a stimulus or experimental driven shift. To provide a baseline to which the effects of adaptation could be compared, we averaged participant judgments across the pre- and postadaptation tests.

**Fig. 5:**
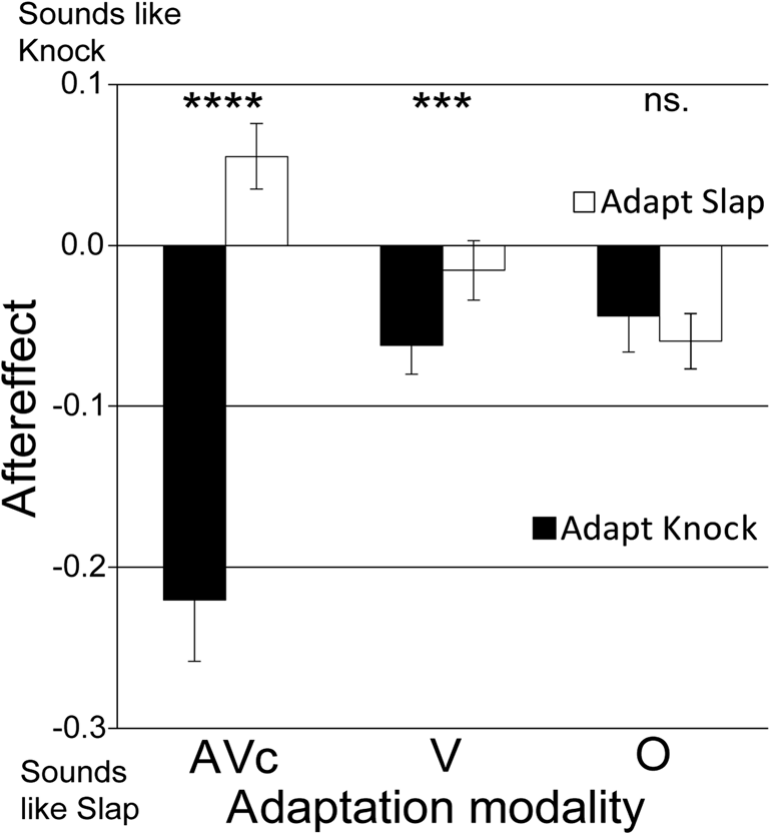
Adaptation to visual actions, but not action words, biases action sound perception. Ambiguous action sound perception following adaptation to knocks (*dark bars*) and slaps (*white bars*). Adapting stimuli were presented as congruent audiovisual stimuli (AVc), as visual stimuli alone (V), or as orthographically presented action words (O). *Error bars* denote ±*SEM. Asterisks* denote a significant difference between action conditions for each adaptor modality. ****p < .001. ***p < .005

As in Experiment 3, the adapting action had a significant effect on the perception of action sounds (see Fig. 5), *F*(1, 19) = 42.17, *p* < .001, η_p_^2^ = 0.69, 95 % CIs adapt knock [−.15, −.06], adapt slap [−.04, .02]. The modality of the adaptor also had a significant influence on the magnitude of the aftereffects (Adapting Action × Modality interaction), *F*(1.32, 25.10) = 32.58, *p* < .001, η_p_^2^ = 0.63, CIs adapt knock AV [−.30, −.14], V [−.10, −.02], O [−.09, .00], adapt slap AV [.01, .10], V [−.05, .02], O [−.09, −.02], Greenhouse–Geisser correction applied. There was no main effect of modality, *F*(2, 38) = 3.06, *p* = .059, η_p_^2^ = 0.14, CIs AV [−.13, −.04], V [−.07, .00], O [−.09, −.01]. Audiovisual (AV) adaptors generated large and significantly different aftereffects (*M* = .27, *SD* = .18), *t*(19) = 6.70, *p* < .001, Cohen’s *d* = 2.11, *r* = .73, CI [.19, .36], B > 1,000, visual adaptors generated smaller, but significantly different aftereffects (*M* = .05, *SD* = .06), *t*(19) = 3.47, *p* = .003, Cohen’s *d* = .57, *r* = .28, CI [.02, .07], B = 40, while adapting to action words did not result in significantly different aftereffects (*M* = .01, *SD* = .08), *t*(19) = .88, *p* > .250, Cohen’s *d* = .18, *r* = .09, CI [−.02, .05], B = .15. Bayes factors indicated evidence for auditory aftereffects generated by audiovisual and visual adaptors, but not for orthographic adaptors. Instead, the Bayes factor for the orthographic control provides support of the null hypothesis: that there is no difference in the aftereffects induced by the different adapting words. Although adaptation to both knock and slap action words appear to generate small slap aftereffects, the knock aftereffect was not significant (*M* = −.04, *SD* = 1.0), 1-sample *t* test: *t*(19) = 1.9, *p* = .067, Cohen’s *d* = .61, *r* = .29, CI [−.09, .00], B = 1.19, and the slap aftereffect, although significant, was not in the direction predicted (*M* = −.06, *SD* = .08), 1-sample *t* test: *t*(19) = 3.5, *p* = .003, Cohen’s *d* = 1.1, *r* = .48, CI [−.09, .02], B = .06. Bayes factors indicated that there was neither evidence for, nor against, the knock word adaptor generating an aftereffect, while there was clear evidence against the slap word adaptor generating an aftereffect.

## Discussion

The results of Experiment 1 show that adaptation to auditory, visual, or audiovisual hand actions can enhance the discriminability of subsequent auditory hand actions. Experiment 2 showed that visual adaptation-induced enhancement of auditory action discrimination was greatest when the adapting visual action matched the test auditory actions. Although previous research has shown that visual adaptation can improve visual discrimination around the adaptor (e.g., Clifford & Langley, 1996; Keefe et al., 2013; Phinney et al., 1997), here we show that visual adaptation improves auditory discrimination around the adaptor. In Experiments 3 and 4, we found that adaptation to the visual, auditory, and audiovisual actions can also generate auditory aftereffects where perception of subsequent ambiguous auditory test stimuli are biased away from the adapted stimulus. These crossmodal aftereffects occurred following observation of silent videos of hand actions, but not following observation of action words.

In Experiment 1, although the greatest enhancement in auditory discrimination was seen with the auditory adaptor, there was no significant difference between the auditory, audiovisual, and visual conditions. We predicted that the audiovisual adaptor would have the greatest effect on discriminability, given that when sight and sound are presented together, perception is typically enhanced (e.g., Fort et al., 2002; Giard & Peronnet, 1999), and we might have expected the effect of adaptation to be greatest under this condition. Furthermore, in a previous study using auditory, visual, and audiovisual adaptors (Kitagawa & Ichihara, 2002), aftereffects were greatest following audiovisual rather than unimodal adaptation. However, such audiovisual enhancement was not observed here. One possible explanation for the similarity between the effects observed with different modality adaptors might be that the adaptation-induced enhancement had reached saturation under all conditions. Our experiment was designed to maximize our chance of finding, potentially small, crossmodal effects of adaptation, by including both an initial adaptation phase and repeated top-up adaptation on every trial, to maintain a high state of adaptation. Thus, the particular techniques we used here may have resulted in adaptation reaching a ceiling with auditory adaptors, and thus masked any potential multimodal enhancement of adaptation that might be observed if we had used less effective auditory adaptors.

One possibility is that the enhancements observed in Experiment 1 were not because of perceptual adaptation but rather attributed to a generalized enhancement in auditory processing due to temporal cueing during the adaptation blocks of testing (Bausenhart, Rolke, & Ulrich, 2007). For example, it might be argued that top-up adaptation of eight repeats could induce a regular and predictable temporal structure to the trial, thereby reducing temporal uncertainty for the presentation of the subsequent test stimuli and thus increasing sensitivity to the action sounds. However, we believe this explanation to be unlikely. First, we use a fixation cross in both no-adaptation and adaptation conditions to cue the observer about the upcoming auditory test stimuli, and thus cueing of participants’ attention was similar under all conditions. Second, under the no-adaptation condition, the temporal structure of the experiment is likely to be more apparent given the more frequent occurrence of trials during blocks of testing; however, in the no-adaptation condition auditory discrimination was poorest. Finally, in Experiment 2, we found a significant difference between adaptation-induced discrimination dependent upon whether the adapting action matched the test actions, even though the temporal structure of the trials and participant cuing was identical in both conditions.

Discrimination was most enhanced when the adapting visual action “matched” the auditory action (i.e., the adapting and test actors were conceptually similar), and these results are commensurate with previous unimodal results that show that adaptation enhances discrimination around the adaptor but not for dissimilar stimuli (e.g., Chen et al., 2010; Keefe et al., 2013; Rhodes et al., 2007). Adaptation to nonmatching visual actions, however, resulted in some auditory action enhancement, suggesting that there may be potential overlap in the representation of the knock and slap actions. For example, single units in the monkey superior temporal sulcus respond with varying degrees of selectivity to different hand actions. While some cells will respond to one hand action, most also show a degree of sensitivity to another hand action with a different goal (e.g., see Barraclough et al., 2009).

The crossmodal enhancements in auditory action discrimination we observed in both Experiments 1 and 2 were quite small. Changes in the JNDs induced by adaptation were in the region of 1–2 % of the blend between the two different hand action sounds. The effect of visual adaptation was to reduce the auditory discrimination threshold by 22 % in Experiment 1, and 30 % in Experiment 2. The magnitude of the adaptation-induced crossmodal enhancements in discrimination we observed, however, are commensurate with other small enhancements in discriminability observed following adaptation by same modality stimuli (e.g., Chen et al., 2010; Keefe et al., 2013; Yang et al., 2011). The small effects we observed occurred over a relatively short period (~30 s). Although not examined directly here, we would expect that increases in sensitivity would be proportional to the duration of adaptation, thus we would see greater improvements in action sound discrimination over longer periods as might be expected under real world viewing conditions (Clifford & Langley, 1996).

Adaptation to visual actions also generated small, but significant, action sound aftereffects, where subsequent auditory actions sounded less like the adapting visual action. These repulsive aftereffects observed in Experiments 3 and 4 were more akin to previous measures of crossmodal aftereffects (e.g., Hills et al., 2010; Skuk & Schweinberger, 2013; Zaske et al., 2010). Aftereffects only occurred following adaptation to visual movies of actions, and there was no evidence for aftereffects being induced by adaptation to the names of the actions, suggesting that shifts in the categorization of action sounds are perceptual, rather than conceptual, in nature (cf. Schweinberger et al., 2008). Orthographic slap adaptation resulted in a small but significant effect that was reminiscent of priming (Tulving & Schacter, 1990) rather than adaptation, as the auditory actions sounded more like the action named. However, we believe this effect is likely due to an overall shift in the baseline during Experiment 4 as the preadaptation control stimuli were initially judged as being more like knock actions.

However, the aftereffects observed in Experiments 3 and 4, unlike the enhancements in discrimination measured in Experiments 1 and 2, fall foul of the arguments described by Morgan et al. (2013) and Storrs (2015). Only the method employed during Experiments 1 and 2, where we found crossmodal adaptation-induced enhancement in auditory discrimination, can distinguish a perceptual basis for crossmodal adaptation from a potential postperceptual response bias. In Experiments 1 and 2, we measured participants’ sensitivity to action sounds by assessing the shape of the psychometric function rather than a shift in the position of the psychometric function (i.e., the point of subjective equality, or PSE). Shifts in the PSE of the psychometric function can be achieved by observers voluntarily (Morgan et al., 2012) without altering the slope, showing that measures of aftereffects derived from such PSE shifts are open to observer bias (see also Morgan, 2014). In contrast, perceptual sensitivity, as we measured in Experiments 1 and 2, is not confounded with decision-making criteria and thus demonstrates a perceptual basis for the crossmodal aftereffects we observed. Nevertheless, our final two experiments indicate that the stimuli used to demonstrate perceptual enhancements around the adapted level can also generate perceptual aftereffects when using a different methodology.

We tested not only the effect of visual adaptation but also the effect of auditory and audiovisual adaptation on auditory aftereffects. Given previous demonstrations of the effectiveness of multimodal stimuli over unimodal stimuli (e.g., Kitagawa & Ichihara, 2002), it was surprising that we did not see greater aftereffects with congruent multimodal stimuli. However, as for Experiment 1, our experiment was designed to maximize the chance of finding small crossmodal aftereffects, and saturation of the aftereffect may have ensued. If this was the case, the addition of the visual component in the AVc condition would have had little influence on aftereffect magnitude, as we observed. Kitagawa and Ichihara (2002) also showed a decrease in the magnitude of their aftereffects when the auditory adaptors were presented concurrently with incongruent visual stimuli (AVi). We might have expected a similar effect. Although the aftereffects generated by the AVi adaptors were smaller than those generated by the AVc adaptors, they were not significantly different (Adapting Action [knock, slap] × Modality [AVc, AVi] interaction): *F*(1, 16) = 1.9, *p* = .187, η_p_^2^ = 0.11, CIs adapt knock AVc [−.29, −.11], AVi [−.26, −.03], adapt slap AVc [.03, .18], AVi [.03, .16]. We attribute this lack of significant reduction in aftereffect magnitude to the dominating influence of the auditory adaptor over the less effective visual adaptor. Multisensory effects are typically inversely related to the effectiveness of both unimodal stimuli (Meredith & Stein, 1986; Stein & Wallace, 1996). Thus, significant decreases in crossmodal aftereffects with incongruent visual stimuli (and indeed, significant increases with congruent visual stimuli) may result from adaptation when the auditory adaptor is less effective than we used here (e.g., by using quieter or masked auditory stimuli).

Finally, action adaptation appears to have two different effects on the perception of auditory actions: an enhancement of action discrimination as well as a bias in action categorization. These two effects could result from, respectively, adaptation-induced processes of decorrelation and self-calibration, seen in neurons in the early visual system (Benucci, Saleem, & Carandini, 2013), occurring in action selective audiovisual neurons later in perceptual processing (e.g. Barraclough, Xiao, Oram, & Perrett, 2005; Kohler et al., 2002). Neurons within the STS of the monkey respond selectively to the hand actions of other individuals (Chitty, Perrett, Mistlin, & Potter, 1985a, 1985b; Perrett et al., 1989), and many of them integrate the specific visual and auditory information about the action itself (Barraclough et al., 2005). Furthermore, the population of cells within the STS appear to represent actions contiguously across a parametric action space (Barraclough et al., 2009; Vangeneugden, Pollick, & Vogels, 2009), while action-sensitive cells in the STS are also susceptible to adaptation (Kuravi, Caggiano, Giese, & Vogels, 2016). Thus, adaptation-induced changes in activity within a population of neurons of these types may underlie the two effects we observe in this study. First, action adaptation may optimize the dynamic range of neurons to reduce redundancy in their representation of the multisensory environment (Barlow & Foldiak, 1989; Clifford et al., 2000), resulting in the enhanced discrimination we observed. Second, prior exposure to different actions may enable the neural representation of multimodal actions to change itself in response to the prevailing statistical characteristics of the multimodal social environment, resulting in a short-term self-calibration and the perception of aftereffects (see Clifford et al., 2000).

In conclusion, we have shown that adaptation to auditory, audiovisual, and visual actions can selectively enhance action sound discrimination, thereby allowing us to detect smaller changes in action sounds. We believe this is the first demonstration of crossmodal adaptation that cannot be explained by possible postperceptual response biases. These effects of adaptation appear to occur over a relatively short period (in the order of minutes) and might represent a mechanism by which our perceptual system optimally calibrates itself to our dynamic multimodal social environment.

## Electronic supplementary material

Below is the link to the electronic supplementary material.

ESM 1 (DOCX 43 kb)

ESM 2 (WAV 117 kb)

ESM 3 (WAV 117 kb)

ESM 4 (WAV 117 kb)

ESM 5 (WAV 117 kb)

ESM 6 (WAV 117 kb)

ESM 7 (WAV 117 kb)

ESM 8 (WAV 117 kb)

ESM 9 (WAV 117 kb)

ESM 10 (WAV 117 kb)
