# Supplementary material for: Visual adaptation enhances action sound discrimination
Source: Atten Percept Psychophys. 2016 Sep 7;79(1):320–32. doi: 10.3758/s13414-016-1199-z (PMC5179587; doi:10.3758/s13414-016-1199-z)
Supplement: Supplementary file 1 — (DOCX 43 kb) [file 13414_2016_1199_MOESM1_ESM.docx]

**Supplementary materials**

For Experiments 1 and 2 we predicted that adaptation at the same level of the test stimulus would enhance discrimination of the test stimuli around the adaptor. We did not expect any aftereffects from these experiments, where the test stimuli appear less like the adaptor. As the adaptor was in the ‘centre’ of the presented test stimuli (adapting sounds were the same as the standard test sound), adaptation would not have a systematic effect to repel the test stimuli away from the adaptor. In order to test if adaptation in Experiments 1 and 2 generated shifts in the categorization of action sounds we examined the point of subjective equality (PSE) derived from the fitted psychometric functions for each of the conditions.

In Experiment 1, adaptation did not have a significant influence on the PSEs calculated for knock sounds (*F*(2.00,33.94)=.075, *p*=.93, *η_p_^2^*=.00, 95% CIs no adapt [79.7, 80.6], adapt audiovisual [79.8, 80.7], adapt visual [79.9, 80.5], adapt auditory [79.8, 80.4], Greenhouse-Geisser correction applied). In Experiment 2, adaptation also did not have a significant influence on the PSEs calculated for knock sounds (*F*(1.560,33.79)=1.55, *p*=.29, *η_p_^2^*=.07, 95% CIs no adapt [80.18, 81.20], adapt knock [79.9, 80.7], adapt slap [79.9, 80.7], Greenhouse-Geisser correction applied).
